# Ultrastructural Analysis of Nanogold-Labeled Cell Surface Microvilli in Liquid by Atmospheric Scanning Electron Microscopy and Their Relevance in Cell Adhesion

**DOI:** 10.3390/ijms141020809

**Published:** 2013-10-16

**Authors:** Toshiyuki Murai, Mari Sato, Hidetoshi Nishiyama, Mitsuo Suga, Chikara Sato

**Affiliations:** 1 Department of Microbiology and Immunology, Graduate School of Medicine, Osaka University, 2-2 Yamada-oka, Suita, Osaka 565-0871, Japan; 2 Biomedical Research Institute, National Institute of Advanced Industrial Science and Technology (AIST), Higashi 1-1-1, Tsukuba, Ibaraki 305-8568, Japan; E-Mails: ma-satou@aist.go.jp (M.S.); ti-sato@aist.go.jp (C.S.); 3 Advanced Technology Division, JEOL Ltd., Akishima, Tokyo 196-8558, Japan; E-Mails: hinishiy@jeol.co.jp (H.N.); msuga@jeol.co.jp (M.S.)

**Keywords:** cell adhesion, cell surface, cytoskeleton, microvilli, immuno-electron microscopy, SEM, correlative microscopy, correlative light and electron microscopy (CLEM), ASEM

## Abstract

The adhesion of leukocytes circulating in the blood to vascular endothelium is critical for their trafficking in the vasculature, and CD44 is an important cell surface receptor for rolling adhesion. In this study, we demonstrate the correlative observation of CD44 distribution at the lymphocyte cell surface in liquid by fluorescence optical microscopy and immuno-electron microscopy using an atmospheric scanning electron microscope (ASEM). The ultrastructure of the cell surface was clearly imaged by ASEM using positively charged Nanogold particles. ASEM analysis demonstrated microvilli projections around the cell surface and the localization of CD44 on the microvilli. Treatment of cells with cytochalasin D resulted in a loss of the microvilli projections and concomitantly abrogated CD44-mediated adhesion to its ligand hyaluronan. These results suggest the functional relevance of microvilli in CD44-mediated rolling adhesion under shear flow.

## 1. Introduction

The adhesion of circulating leukocytes in the blood to endothelium at the sites of inflammation is critical for their subsequent extravasation through the blood vessel wall into the sites of inflammation. The major interaction in the initial recognition is rolling adhesion, which is the primary interaction of leukocytes with vascular endothelial cells under shear flow. Rolling adhesion requires specialized adhesion receptors on the surface of leukocytes. Rolling has been attributed to members of the selectin family proteins [1], but it is also mediated by CD44, a cell surface glycoprotein expressed in various cell types, including lymphoid cells [2]. CD44 is a transmembrane adhesion receptor that has been associated with various biological processes, including leukocyte trafficking and tumor metastasis [3]. By changing its binding ability to the ligand hyaluronan, its adhesive function could be tightly regulated [4–7].

Leukocytes display pointed projections of the plasma membrane, microvilli, on their cell surface, and these structures may facilitate tethering and rolling of leukocytes along the vascular endothelium under flow [8]. Optical microscopy (light microscopy) has been the most widely used method to observe the distribution of receptor molecules at the cell surface by labeling with fluorescence dye. However, conventional optical microscopy is not suitable to observe the ultrastructure, including that of microvilli, due to diffraction-limited resolution. Super-resolution techniques surpass the limits of optical diffraction in light microscopy, and the objects that are less than 100 nm apart can be resolved by these techniques [9–11]. However, the resolution is still insufficient for the observation of fine subcellular surface structures and the distribution of membrane receptors at the cell surface, which requires high spatial resolution of around 10 nm. Leukocyte microvilli have been observed by transmission electron microscopy and scanning electron microscopy (SEM) [12–16] under vacuum after dehydration processes. These observations revealed that the localization of adhesion molecules such as L-selectin to microvilli results in concentration of the receptor, making it accessible to their ligands on endothelial cells [13–16]. However, these conventional electron microscopy techniques are not compatible with wet specimens and require elaborate specimen preparation processes, including dehydration, which may affect delicate subcellular structures.

Several methods have been developed for the observation of wet samples including biological specimens and cells with high resolution, using electron beams. Electron microscopy using environmental capsule [17] enables the direct observation of wet specimens but with small sample volumes [18–21]. Electron beam excitation assisted optical microscopy (EXAM) enables the observation of wet samples [22]. Recently, atmospheric scanning electron microscopy (ASEM) was developed for direct observation of wet specimens in an open dish, which facilitates cell culture and various kinds of labeling [23]. ASEM was applied for the observation of dynamic phenomena of inorganic materials and electrochemical reactions in liquid [24]. ASEM can be used to observe biological specimens such as mammalian cells [25,26], small bacteria [27], and protein crystals [28] in an atmospheric environment without dehydration. The introduction of ASEM in cell biology has revolutionized the observation of samples by enabling direct SEM examination in aqueous environment [25,26]. ASEM also enables correlative observation with light microscopy and electron microscopy [23,25]. Correlative light and electron microscopy (CLEM) has become increasingly important in the analysis of cellular structure and function [29,30].

In the present study, we report the correlative microscopic observation of lymphocytes in water using nanometer-sized gold particles (Nanogold) by ASEM with immuno-fluorescence optical microscopy and immuno-electron microscopy for analysis of surface distribution of CD44, and also report the ultrastructural analysis of cell surface microvilli by using positively charged Nanogold. We also estimated the functional relevance of microvilli to CD44-dependent cell adhesive properties under shear flow.

## 2. Results and Discussion

### 2.1. Correlative Observation of CD44 Distribution on Lymphocyte Cell Surface by ASEM

**Figure 1:**
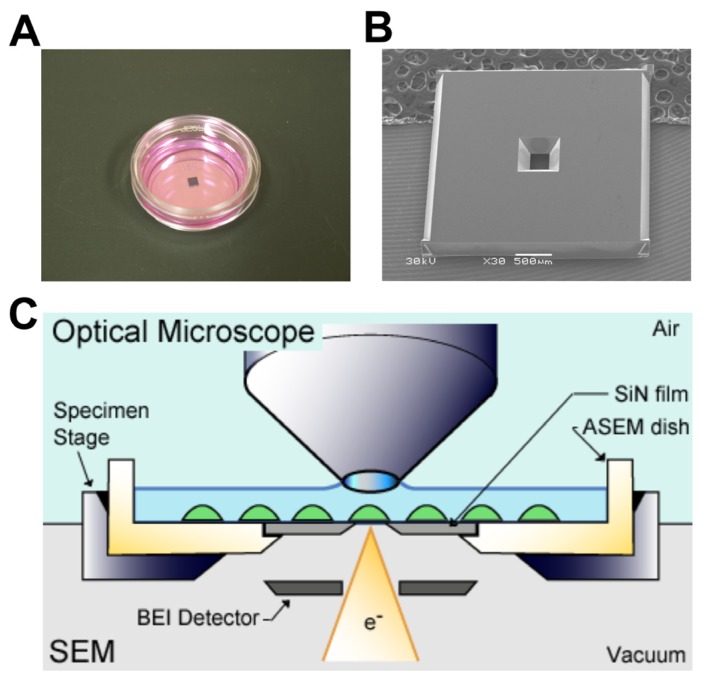
Correlative light and electron microscopy (CLEM) by atmospheric scanning electron microscope (ASEM). (**A**) ASEM dish. A removable 35-mm ASEM dish has a 100-nm silicon nitride (SiN) film window separating vacuum and atmosphere; (**B**) SEM image of an SiN window. Scale bar, 500 μm; and (**C**) Schematic diagram of the ASEM system. An optical microscope capable of fluorescence imaging, is arranged above the inverted SEM, with the specimen dish between them. The removable 35-mm ASEM dish features an SiN film window in its bottom plate, which separates vacuum and atmosphere. The electron beam is projected from underneath onto the cells through the SiN film, and backscattered electrons are captured by the backscattered electron imaging (BEI) detector. The axes of both microscopes are mechanically aligned, and the specimen stage can be shifted two-dimensionally on the *X*–*Y* plane.

Imaging analysis using fluorescence dye is a powerful approach to study cellular distribution of membrane receptors. A limitation of this method is that it lacks fine structural information, which can be overcome using immuno-electron microscopy. For correlative observation with fluorescence optical microscopy and SEM of CD44 distribution at the cell surface, BW5147 T lymphocytes were cultured in an ASEM dish (Figure 1A).

The ASEM dish has a silicon nitride (SiN) window, a thin layer of SiN (100-nm thick), which is transparent against an electron beam (Figure 1B). The window was fabricated by etching the Si side of an Si–SiN bilayered plate made by a chemical vapor deposition (CVD) process on an Si slab [31]. The thickness of the SiN film is a key factor for the spatial resolution of ASEM, and a 100-nm film was found to be the most appropriate as the standard usage by ASEM, considering the safety factor for a rupture of the film. The chip with an SiN window was attached to a polystyrene dish with the SiN side facing up. The ASEM dish was set to a holder of ASEM. In ASEM, the axes of optical microscopy and SEM are aligned to allow concurrent observation in the same areas (Figure 1C). The fluorescence excitation is from above through an objective lens, and the electron beam is projected from underneath onto the specimens. The detectors for optical microscopy and SEM are a Neo sCMOS camera with 2544 × 2160 pixels and a disk-shaped BEI detector, respectively.

**Figure 2:**
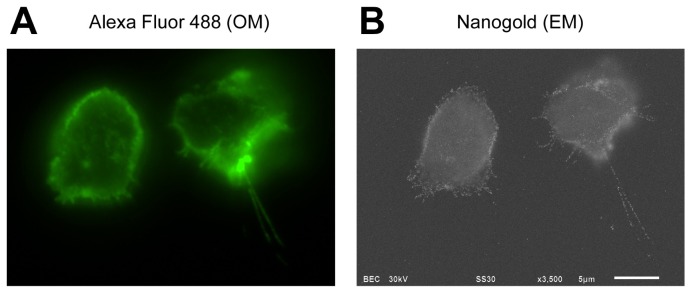
Distribution of CD44 on the plasma membrane, visualized by correlative optical and electron microscopy using ASEM. BW5147 T lymphocytes were labeled with an anti-CD44 monoclonal antibody IM7.8.1, and further with a secondary antibody conjugated both with Alexa Fluor 488 and Nanogold. (**A**) Fluorescence optical microscopy (OM) image; and (**B**) Electron microscopy (EM) image of the same cells after gold enhancement. Signals were clearly observed on the cell body as well as on the microvilli. ASEM images were captured at 3500× magnification. Scale bar represents 5 μm.

To observe CD44 distribution at the cell surface, dual labeling was employed using an anti-CD44 monoclonal antibody and a secondary antibody conjugated with Alexa Fluor 488 and 1.4-nm Nanogold. Apparently entire cell surface with many small protrusions was stained with fluorescence under optical microscopy (Figure 2A). We used normal rat IgG as a staining control, and no signal was detected under optical microscopy, confirming that the cell surface labeling was specific for CD44 (data not shown). In contrast, after gold enhancement, microvilli were clearly observed around the cell surface using ASEM (Figure 2B). These results clearly indicate that CD44 was localized over the entire cell surface including the microvilli. As viewed from the periphery to the center of the cell, signals in SEM image were blurred and diminished due to the scattering of electron beams. The observable area was within the thickness of 2–3 μm from the SiN film. The maximum spatial resolution of ASEM was estimated to be 8 nm, according to the measured distance between two distinguishable gold particles [23]. Recently, de Jonge and colleagueshave reported that epidermal growth factor receptors in intact cells were imaged by environmental SEM with a scanning transmission electron microscopy detector using gold particles, and yielded a high resolution surpassing ASEM [32].

### 2.2. Disruption of Cytoskeletal Structure with Cytochalasin D Abrogates Microvilli Formation and CD44 Activity

**Figure 3:**
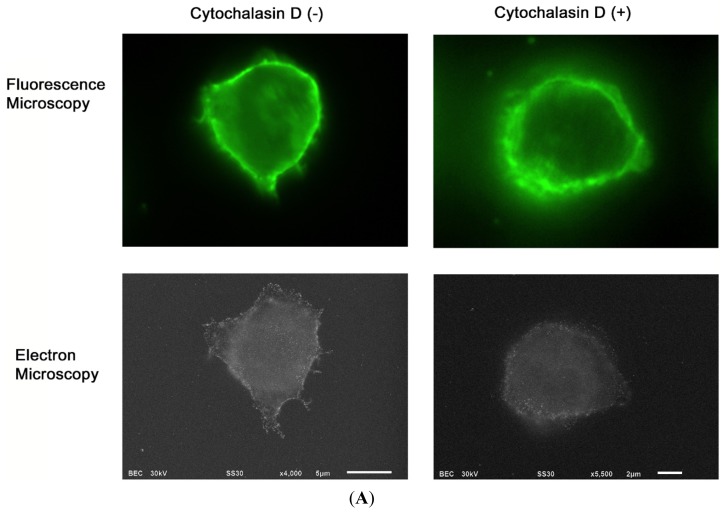

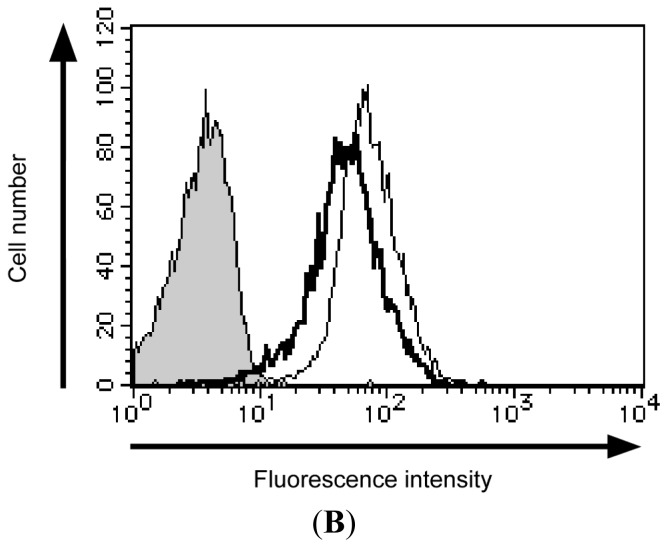
The effect of cytochalasin D on CD44 distribution and activity. (**A**) BW5147 T lymphocytes were left untreated (**left panels**) or treated with 10 μM cytochalasin D on ASEM dishes for 1 h (**right panels**), and fixed with 4% paraformaldehyde. CD44 on the cell surface was labeled with FluorNanogold. The labeled cells were observed by fluorescence microscopy (**upper panels**) or electron microscopy (**lower panels**) after gold enhancement using ASEM at 4000× (**lower left panel**) and 5500× (**lower right panel**) magnifications. Scale bars represent 5 μm (**left panel**) and 2 μm (**right panel**); and (**B**) The effect of cytochalasin D on hyaluronan-binding ability. BW5147 T lymphocytes were treated with 10 μM cytochalasin D (**thick line**) or left untreated (**thin line**) for 1 h at 37 °C, and the extent of FITC-conjugated hyaluronan binding was determined by flow cytometry. **Gray filled profile**, unstained control.

Treatment of cells with cytochalasin D resulted in a loss of surface projections (Figure 3), demonstrating that the microvilli structure required an actin-based cytoskeleton (Figure 3A). Flow cytometric analysis revealed that the treatment with cytochalasin D also reduced the cell’s binding of hyaluronan, a major ligand to CD44 (Figure 3B).

### 2.3. Ultrastructure of Lymphocyte Cell Surface Visualized by Positively Charged Gold Particles

**Figure 4:**
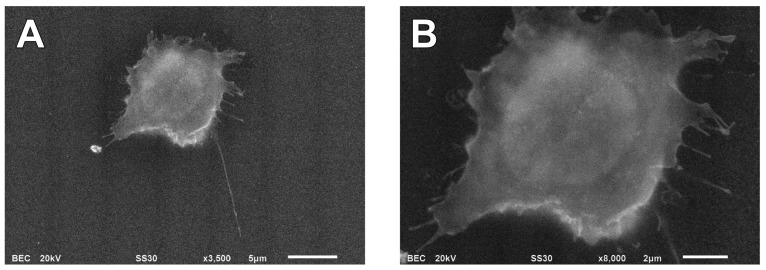
ASEM images by positively charged Nanogold labeling. (**A**) Cells cultured on the ASEM dish were fixed, stained with positively charged Nanogold, and treated with GoldEnhance-EM. 3500× magnification. Scale bar, 5 μm; and (**B**) The cell body was further magnified at 8000× magnification. Scale bar, 2 μm.

To observe the cell surface ultrastructure, cell surface was stained with positively charged Nanogold followed by gold enhancement. Positively charged Nanogold binds to the cell surface by associating with negatively charged moieties of glycans, proteins, and phospholipids present on the cell membrane. Thus, positively charged Nanogold is suitable for staining the cell surface for ultrastructural analysis using ASEM. The surface structure of a whole cell in liquid was clearly observed without dehydration using ASEM at low magnification (Figure 4A). At a higher magnification of 8000×, microvilli projections were clearly observed around the cell (Figure 4B). An estimate of the electron dose at a higher magnification of 8000× was 0.5 e^−^/Å^2^, which is an order of magnitude smaller than the standard dose of 20 e^−^/Å^2^ for single particle studies by cryo-electron microscopy [33]. These results demonstrate that the positively charged Nanogold staining is a reliable method for observing cell surface structure of mammalian cells as well as platinum blue staining [34,35].

### 2.4. Disruption of Cytoskeletal Structure with Cytochalasin D Abrogates CD44-Mediated Rolling under Flow Conditions

**Figure 5:**
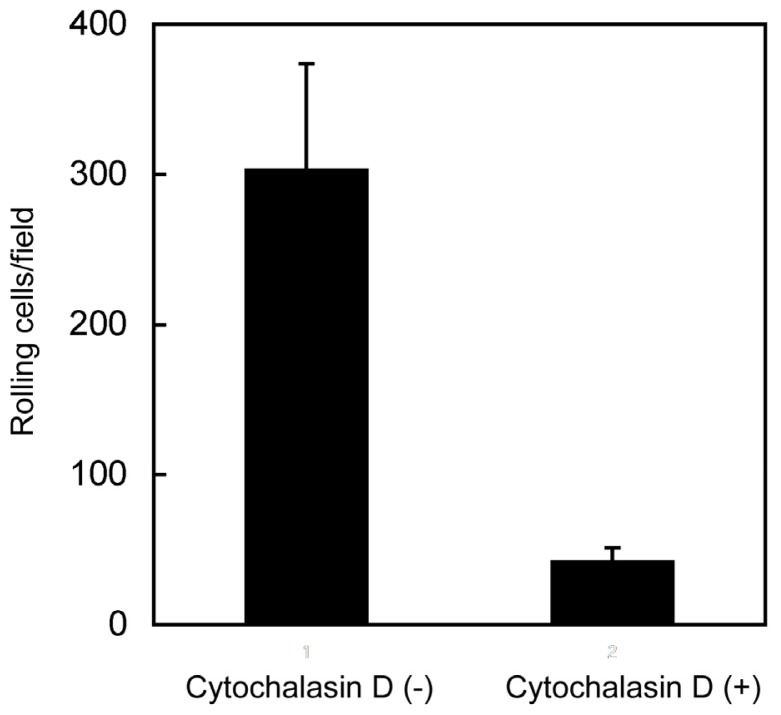
Disruption of the cytoskeletal structure with cytochalasin D abrogates CD44-mediated rolling adhesion under flow conditions. BW5147 T lymphocytes were left untreated or treated with cytochalasin D (10 μM for 1 h), and were then applied continuously to capillary tubes whose inner surface had been coated with hyaluronan. The number of rolling cells at wall shear stress of 1.2 dyn/cm^2^ was determined as described in the Experimental Section.

Cytochalasin D, which facilitates depolymerization of actin filaments, decreased rolling on hyaluronan (Figure 5). Because the CD44’s function and the rolling ability of cells were affected by membrane microenvironment [25,26,36], further examination of the involvement of membrane structure may be necessary for elucidation of the functional relevance of microvilli in CD44-mediated cell adhesion under shear flow.

## 3. Experimental Section

### 3.1. Reagents

Rat anti-mouse CD44 monoclonal antibody IM7.8.1 was purchased from BioLegend (San Diego, CA, USA). Fab’ fragment of Nanogold (1.4 nm)- and Alexa Fluor 488-conjugated goat anti-rat IgG (FluorNanogold) and GoldEnhance-EM were purchased from Nanoprobes, Inc. (Yaphank, NY, USA). Cytochalasin D was purchased from Sigma-Aldrich (St. Louis, MO, USA).

### 3.2. Cell Culture

The mouse T lymphocyte cell line BW5147, obtained from American Tissue Culture Collection (ATCC, Manassas, VA, USA), was maintained in RPMI 1640 medium (Sigma-Aldrich) supplemented with 10% fetal calf serum, 100 units/mL penicillin, and 100 μg/mL streptomycin, and incubated at 37 °C in an atmosphere containing 5% CO_2_.

### 3.3. Cell Staining

Immunolabeling with gold conjugates and imaging with ASEM were performed as described previously [25]. In brief, cells were cultured on an SiN film of 100 nm in thickness, in RPMI supplemented with 10% fetal calf serum, in a 5% CO_2_ atmosphere at 37 °C. The cells were untreated or treated with 10 μM cytochalasin D for 1 h and fixed with 4% paraformaldehyde in phosphate-buffered saline (PBS) at room temperature for 10 min. For detecting CD44 on the cell surface, the cells were incubated with 1% skim milk/PBS for 30 min, with IM7.8.1 antibody for 1 h, and then with Fab’ fragment of Nanogold- and Alexa Fluor 488-conjugated goat anti-rat IgG for 30 min. After fixation with 1% glutaraldehyde for 15 min at room temperature, the Nanogold signal was enhanced using GoldEnhance-EM at room temperature for 5 min.

For positively charged Nanogold labeling, glutaraldehyde-fixed cells were incubated with 3 μM positively charged Nanogold solution (Nanoprobes) for 20 min at room temperature. After washing with double distilled water (DDW), the size of the gold particles was increased by gold enhancement using GoldEnhance-EM #2133 (Nanoprobes). Protocol #2113 was employed, but steps #3 and #4 (addition of 50 mM glycine in PBS to inactivate residual paraformaldehyde) were omitted, and the development time was 10 min, followed by washing with DDW.

### 3.4. ASEM

The configuration of the ClairScope, JASM-6200 (JEOL Ltd., Tokyo, Japan) is illustrated in Figure 1. The electron beam of the inverted SEM is projected from underneath (in a vacuum) through the SiN film of the ASEM dish onto the specimen stage, which is at atmospheric pressure. Except for this SiN window, the ASEM dish is the same (material and size) as the polystyrene Petri dishes used for cell culture. It holds approximately 3 mL of medium and can be removed from the microscope and used for the prolonged culture of various types of cells in a CO_2_ incubator. Thus, specimens can later be imaged *in situ* by SEM, with the backscattered electrons being captured by a backscattered electron imaging (BEI) detector (Figure 1C). Fluorescence images can be captured with a Neo sCMOS camera with 2544 × 2160 pixels (Andor Technology, Belfast, UK).

The inverted SEM of the ClairScope was operated at 30 kV for immuno-labeling and 20 kV for positively charged gold labeling. All specimens were imaged in 10 mg/mL dextrose in DDW. Cells were fixed and stained *in situ* beforehand as required.

### 3.5. Flow Cytometry

To measure hyaluronan binding, cells were incubated on ice with or without 2 μg/mL FITC-conjugated hyaluronan (PG Research, Tokyo, Japan) for 1 h. Samples were analyzed using a FACS Calibur (BD Biosciences, San Jose, CA, USA) with FlowJo software (Tree Star, Ashland, OR, USA).

### 3.6. Shear Flow Assay

The shear flow assay was performed based on the method as previously described [26]. BW5147 T lymphocytes that had been subjected to cytochalasin D treatment or were left untreated, were rinsed and resuspended in prewarmed RPMI 1640 medium at 1 × 10^6^ cells/mL. The cell suspension was then transfused through a capillary tube (Drummond Scientific, Broomall, PA, USA), the inner surface of which had been coated with 0.1 mg/mL NeutrAvidin (Molecular Probes, Eugene, OR, USA) and subsequently with 25 μg/mL biotin-conjugated hyaluronan (Hyalose, Oklahoma, OK, USA), at a wall shear stress of 1.2 dyn/cm^2^ using a syringe pump (Harvard Apparatus, South Natick, MA, USA). The rolling cells were observed under an inverted phase-contrast microscope with a 10× objective, and analyzed using ImageJ software (NIH, Bethesda, MD, USA).

## 4. Conclusions

In the present study, we report the ASEM observation of the cell surface ultrastructure of lymphocytes in an aqueous environment using nanometer-sized gold particles. The ASEM analysis clearly demonstrated the microvilli projection around the cell surface, and the localization of CD44 on the microvilli. The results presented in this paper suggest that the functional relevance of microvilli in CD44-mediated rolling adhesion under shear flow. ASEM is a powerful tool for ultrastructural analysis of biological samples, and the method demonstrated in this paper can be effectively applied to studies on cellular structure and function.
